# Molecular Evaluation of the Mating Dynamics of Snow Crab *(Chionoecetes opilio)* in the Eastern Bering Sea

**DOI:** 10.1002/ece3.70416

**Published:** 2024-10-27

**Authors:** Laura M. Slater, William Gaeuman, Wei Cheng, Gordon H. Kruse, Christopher Habicht, Douglas Pengilly

**Affiliations:** ^1^ College of Fisheries and Ocean Sciences University of Alaska Fairbanks Juneau Alaska USA; ^2^ Division of Commercial Fisheries Alaska Department of Fish and Game (ADF&G) Kodiak Alaska USA; ^3^ Division of Commercial Fisheries, Gene Conservation Laboratory ADF&G Anchorage Alaska USA

**Keywords:** fishery management, hybrids, mating, paternity, reproduction, snow crab

## Abstract

Snow crab (*Chionoecetes opilio*) in the eastern Bering Sea (EBS) supports a valuable crab fishery that harvests large males. To better understand the potential impact of the presence of snow‐Tanner hybrids (*C. opilio* × *C. bairdi*) on snow crab mating dynamics, the maternal lineage of hybrids was evaluated using single nucleotide polymorphisms (SNPs). Contrary to results from a previous study that indicated hybrids result only from crosses between Tanner crab (*C*. *bairdi*) males and snow crab females, results from this study showed hybrids result from bidirectional parental crosses. SNP and microsatellite markers were used to evaluate the species and number of males detected as mates in female sperm reserves acquired during mating and as sires among embryos in brooded clutches. The incidence of interspecies mating between female snow crab and Tanner crab or hybrid males was low (2%), suggesting interspecies mating is not currently a pressing fishery management concern. Most females had stored sperm from either a single mate (59%) or two mates (32%), which reflects fewer mates than observed for other snow crab populations. Few females were observed with either no stored sperm (5%) or sperm from three to four mates (4%). Single (82%) or dual (18%) paternity was found among embryos in brooded clutches. Sperm from some mates appeared to be fully utilized for fertilization of the brooded clutch for 35% of females. In contrast to findings for other snow crab populations, no significant differences were detected in the numbers of mates or sires between primiparous and multiparous females. The low extent of polyandry observed may suggest that female snow crab in the EBS have limited mating opportunities, potentially leading to insufficient sperm reserves to fertilize subsequent clutches without remating.

## Graphical abstract

Most snow crab females in the eastern Bering Sea had stored sperm from one or two mates. While single paternity was common, some females had clutches sired by two males. Female snow crab primarily mated with male snow crab, with few instances of interspecies mating.

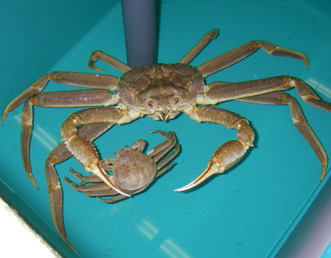

## 1 Introduction

The snow crab (*Chionoecetes opilio*) population in the eastern Bering Sea (EBS) recently supported a large commercial crab fishery of great economic value. During 2016–2020, 59–68 commercial fishing vessels landed 9–15 thousand *t* valued at US $92–132 million annually (Garber‐Yonts and Lee 2022). Annual harvest levels are based on estimates of mature male biomass, which is used as an index of stock reproductive potential. However, little is known about the influence of male‐only harvest on female reproductive output for EBS snow crab. In other crustacean stocks, sex‐specific harvests have been associated with deleterious consequences, including sperm depletion (Pardo et al. 2015; Sato et al. 2005), reduced egg fertilization rate and clutch size (Sainte‐Marie, Sévigny, and Carpentier 2002; Sato et al. 2005), and altered mating behaviors, including reduced mate‐guarding time, smaller ejaculate size, and agonistic interactions resulting from changes in male size composition (Jivoff 2003; Sato and Goshima 2007; Sainte‐Marie et al. 2008; Butler, MacDiarmid, and Gnanalingam 2015). Like many crab stocks, annual recruitment and mature male biomass are highly variable, resulting in “boom‐and‐bust” cycles in fishery harvests, which varied several orders of magnitude from 149 thousand *t* in 1991 to 0 during the 2022/23 fishing season when the fishery was closed due to depressed stock abundance (Nichols, Westphal, and Shaishnikoff 2022). The rapid population decline from 2018 to 2021 has been attributed to starvation resulting from a marine heatwave (Szuwalski et al. 2023).

**FIGURE 1:**
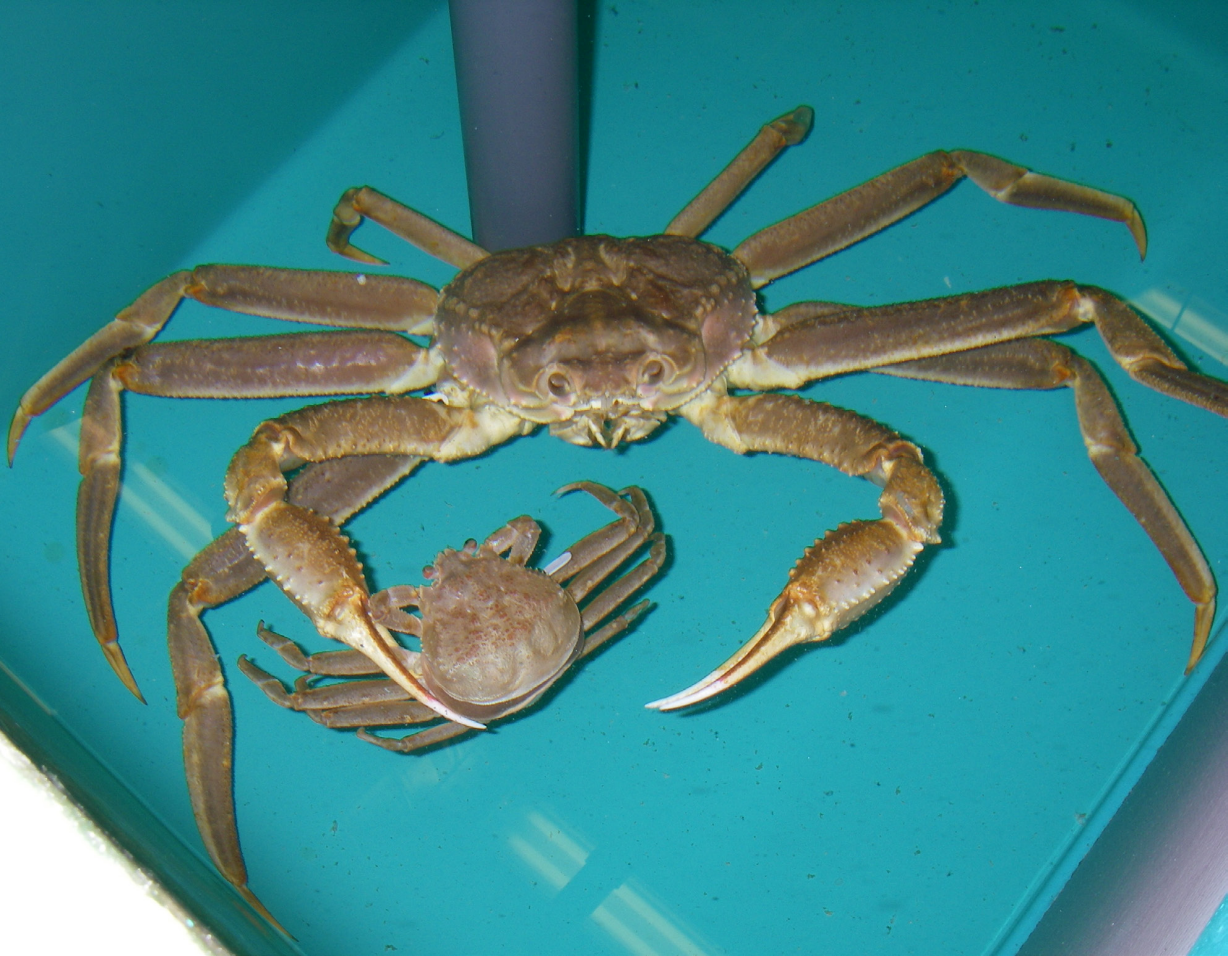
Male snow crab grasping a female snow crab in a mating embrace as she begins her molt to maturity. The female's molting process is evident by the raised carapace, revealing the soft new shell underneath. Photo taken during a mating study at the Alaska Department of Fish and Game seawater laboratory in Kodiak, Alaska (Photo credit: Laura Slater).

Snow crab possess a complex mating system. They display determinate growth, reaching a final size beyond which no further body growth occurs. This “terminal molt” is associated with sexual maturity for females and morphometric maturity for males, which confers advantages in sexual competition and sperm production. Adolescent males, which are functionally mature but have not undergone the terminal molt, are capable of mating (Sainte‐Marie and Lovrich 1994). Adolescent snow crab males are generally excluded from mating by the presence of large adult males in small bay systems in the Gulf of Saint Lawrence, where aggregation occurs during the mating season (Sainte‐Marie et al. 2008). However, adolescent and small adult males may constitute the pool of available mates for females in the middle domain of the EBS, due to the distinct spatial distribution of snow crab by size and sex across the vast EBS shelf (Parada et al. 2010). A primiparous female broods her first clutch after molting to maturity, while a multiparous female broods her second or subsequent clutch. While in a vulnerable soft‐shell condition, primiparous females have little control over mate selection, thus conferring choice (and associated competition) to males (Figure 1). During subsequent mating seasons, females aggregate in mounds that deter male crab, possibly due to excessively high pheromone concentrations (Stevens, Haaga, and Donaldson 1994; Sainte‐Marie et al. 2008). These females can leave the mound to interact with males, and their hard shells allow them to fend off unwanted mates, giving them greater control over mate choice during the mating season.

Like other brachyurans, *Chionoecetes* females possess bilaterally paired internal organs (seminal receptacles) that receive and store sperm acquired during mating. These structures, combined with a polygamous mating system, may serve as safeguards against sperm limitation. When sperm received during mating exceeds sperm expended during egg extrusion, residual stored sperm can be used to fertilize subsequent egg clutches (Sainte‐Marie 1993; Paul 1984). Snow crab mating success is complicated by differential ages of maturation between the sexes resulting in wide swings in adult sex ratios (Sainte‐Marie et al. 2008) and an ontogenetic migration across the EBS continental shelf resulting in spatial separation of large adult males and primiparous females (Zheng, Kruse, and Ackley 2001; Parada et al. 2010).

Fishery management is further complicated by the presence of snow‐Tanner hybrids (*C. opilio* × *C. bairdi*), which exhibit intermediate morphological features between snow crab and Tanner crab (*C*. *bairdi*) (Urban et al. 2002). In early EBS crab surveys, estimates of hybrid abundance were as high as 40% of those for Tanner crab (Reeves 1977). However, later survey reports called into question the accuracy of putative identification of hybrids (Otto, Fukuyama, and Armetta 1980). The current fisheries management approach for hybrids provides a buffer between the snow crab harvest limits and the realized catch. The estimated abundance of hybrids is not considered in determining harvest levels for snow and Tanner crabs, and the strict legal definition of Tanner crab results in harvested hybrid males being counted towards snow crab harvest limits. While interspecies mating is recognized in the EBS, the extent to which it occurs among snow crab females remains unknown. Understanding how frequently snow crab females mate with congeners will help determine the extent to which measures of their reproductive potential reflect snow crab population renewal processes.

Indicators of female reproductive potential that incorporate information on mating success could inform the development of a precautionary approach management system for EBS snow crab, such as the one developed for snow crab in Newfoundland (Mullowney et al. 2024). Previous studies have demonstrated that molecular markers are an effective tool for understanding complex reproductive dynamics in snow crab (Urbani, Sainte‐Marie, et al. 1998). Because female sperm reserves provide a partial record of mating history, molecular analysis of seminal receptacle contents can indicate whether a female crab has mated with multiple males within and across mating seasons and whether she has mated with conspecific or interspecific males. Paternity of embryos in a brooded clutch can provide additional information about a female's mating history, especially in situations where all sperm from a given male is expended in clutch fertilization. The objectives of this study were to: (1) determine the maternal lineage of hybrids; (2) estimate the number and species of mates detected in female sperm reserves; and (3) estimate the number of sires that contributed to paternity of embryos in brooded clutches and determine whether all sire genotypes were present in female sperm reserves. Genetic markers were used to evaluate samples from mature female putative snow crab, Tanner crab, and hybrids collected from the EBS between 2007 and 2018.

## 2 Materials and Methods

### 2.1 Use of Archived Samples

**FIGURE 2:**
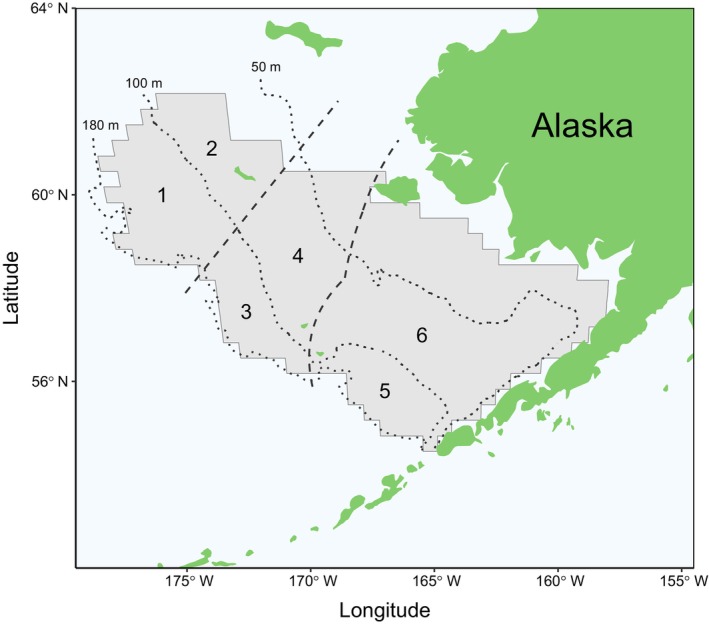
Map of the eastern Bering Sea (EBS) with locations of the six regions used in this study. Regions were defined by the intersection of three areas (northwest, central, and southeast), with boundaries shown by dashed lines, and two oceanographic domains (middle: 50–100 m and outer: 100–180 m), with bathymetric contours shown by dotted lines. Regions are 1: Northwest outer, 2: Northwest middle, 3: Central outer, 4: Central middle, 5: Southeast outer, and 6: Southeast middle. Crabs were collected from the National Oceanic and Atmospheric Administration EBS shelf bottom trawl survey, the extent of which is shown in gray.

Archived samples and associated data for crab specimens collected between 2007 and 2016 were provided by the Alaska Department of Fish and Game (ADF&G) from a long‐term study of reproductive potential of female *Chionoecetes* crabs in the EBS (Laura Slater, unpublished data). Crabs were collected each year from a subset of stations during the National Oceanic and Atmospheric Administration (NOAA) EBS shelf bottom trawl survey in summer months (Figure 2). Stations were sampled following a systematic sampling plan using criteria established for each species. A maximum limit of 15 crab per species per station was established in 2011. Each crab was identified as putative snow crab, Tanner crab, or hybrid based on visual evaluation of morphological features, including carapace shape, epistome shape, and eye color (Jadamec, Donaldson, and Cullenberg 1999; Urban et al. 2002). Because these traits vary along a gradient between the two species, with hybrids occupying an intermediate position, the identification of putative species is subjective. Crabs were transported to the ADF&G seawater laboratory in Kodiak, Alaska, and held live until processing. Crabs that died during transport or holding were either processed soon after death or frozen for later processing.

In the laboratory, crabs were selected haphazardly and processed following methods developed for ADF&G's long‐term study (Laura Slater, personal communication). For all hybrids and a subset of snow and Tanner crabs processed, a portion of the merus segment of one pereiopod (i.e., walking leg) was preserved in 95% denatured ethanol and archived. If both sides of paired seminal receptacles appeared similar during visual examination, the left seminal receptacle was preserved in 95% denatured ethanol for every other snow crab. All merus samples from hybrids, a subset of merus samples from snow and Tanner crabs, and all snow crab seminal receptacles were used in this study. The preservative for these samples was replaced with reagent ethanol. Data included putative species, shell condition (i.e., new shell, old shell), carapace width, and clutch fullness score (i.e., 1: empty, 2: scant, 3: quarter full, 4: half full, 5: three quarters full, and 6: full). Shell condition was used as a proxy for female life history stage based on the assumption that ovigerous new shell females were primiparous and ovigerous old shell females were multiparous. Depending on temperature, embryo development for snow crab in the EBS can extend from 1 year of brooding to 2 (Gardner et al. 2021), meaning a female carrying her first clutch for a second year could be misclassified as multiparous. Data on female sperm reserves were collected from the right seminal receptacle after preservation in 10% buffered formalin for at least a week, including seminal receptacle load (i.e., wet weight of contents) and estimated number of ejaculate layers (Urbani, Sainte‐Marie, et al. 1998). Data also included the estimated age of each ejaculate layer classified as either fresh (the most recent mating season) or old (from a prior mating season), based on visual assessment of color and moisture content (Duluc, Sainte‐Marie, and Brêthes 2005; Bernard Sainte‐Marie, Fisheries and Oceans Canada (DFO), personal communication).

### 2.2 Genetic Markers

Two single nucleotide polymorphisms (SNPs) previously developed to distinguish between EBS snow crab, Tanner crab, and hybrids (Smith, Grant, and Seeb 2005) were used to identify species and maternal lineage. These were a nuclear SNP from the rRNA internal transcribed spacer 1 region (*ITS*) and a mitochondrial SNP from the *16S* rRNA gene (*16S*). Merus samples from a subset of putative snow crab (*n =* 32), Tanner crab (*n =* 32), and hybrids (*n* = 31) collected between 2009 and 2016 were used to evaluate the performance of all markers (hereafter referred to as the evaluation subset). Genomic DNA was extracted from merus muscle tissue using NucleoSpin Tissue (Macherey‐Nagel Inc., Bethlehem, PA). To identify genotypes at the SNP markers, samples were pre‐amplified using polymerase chain reaction (PCR) on the Gene Amp PCR System 9700 thermocycler (Applied Biosystems, Inc., Foster City, CA) and assayed using Taqman Assay (ThermoFisher Scientific, Waltham, MA) following the standard protocol with GTXpress Master Mix in a 5 μL reaction volume. Amplicons were scanned by a QuantStudio 12 K Flex (ThermoFisher Scientific) and scored by TaqMan Genotyper Software (ThermoFisher Scientific). Species identification was determined based on the nucleotides present at the nuclear *ITS* marker as snow crab (both guanine: G/G), Tanner crab (both cytosine: C/C), or hybrid (a combination of cytosine and guanine: C/G). Maternal lineage of hybrids and verification of species identification for snow and Tanner crabs were based on the nucleotides present at the mitochondrial *16S* marker as snow crab (thymine: T) or Tanner crab (cytosine: C). Putative species identifications were confirmed by *ITS* genotypes for most crabs in the evaluation subset (*n* = 92), except one putative Tanner crab identified as a hybrid, one putative hybrid identified as a snow crab, and one putative hybrid excluded due to a genotyping error. During completion of study objectives, putative species identifications were assessed using *ITS* genotypes for hybrids and results from both SNPs when available for snow crab, and otherwise either available SNP.

Eight microsatellite loci developed for snow crab were evaluated with respect to their utility for distinguishing unique male contributors to female sperm reserves and clutch paternity. Candidate loci were *Co17‐nfrdi*, *Co34‐nfrdi*, *Co36‐nfrdi* (Kang, Park, Kim, Ko 2013; Kang, Park, Kim, Choi, et al. 2013), *EC0106* (An, Jeong, and Park 2007), *Cop2*, *Cop77* (Puebla, Parent, and Sévigny 2003), *Cop4‐1*, and *Cop24‐3* (Urbani, Sévigny, et al. 1998). These loci were selected based on their repeat motifs and polymorphic nature as determined by number of alleles.

PCR was used to amplify the eight candidate microsatellite loci in eight snow crab and eight Tanner crab from the evaluation subset on the Gene Amp PCR System 9700 thermocycler (Applied Biosystems, Inc.). Two Taq DNA polymerases, Ex Taq DNA polymerase (Takara Bio USA, Inc., Mountain View, CA) and Dream Taq DNA polymerase (Life Technologies Corporation, Grand Island, NY), were tested. Five of the eight loci were successfully amplified with Ex Taq DNA polymerase (i.e., *Co17‐nfrdi*, *Co36‐nfrdi*, *Cop2*, *Co34‐nfrdi*, and *EC0106*; Table A1 in Appendix A), while none were successfully amplified with Dream Taq DNA polymerase. The five loci that amplified were then optimized using the full evaluation subset, and thermal cycling profiles varied among loci (Table A2 in Appendix A). PCR amplification was carried out in a 10 μL mixture of 2 μL template DNA (~0.1 μg μL^−1^) in 1x ExTaq Buffer (Takara Bio USA, Inc.), 3.0–3.5 mM MgCl_2_ (Promega Inc., Madison, WI), 0.20 mM of each nucleotide (Applied Biosystems, Inc.), 0.40 μM of forward and reverse primers, 2 M Betaine (MilliporeSigma, Inc., Burlington, MA), 0.25 μM Spermidine (MilliporeSigma, Inc.), 100 μg mL^−1^ of BSA (MilliporeSigma, Inc.), 0.05 U ExTaq DNA polymerase, and deionized water. Amplified products were size fractionated in an Applied Biosystems 3730 capillary DNA sequencer and genotyped with GeneMapper 5.0 (Applied Biosystems, Inc.).

Results for the five optimized microsatellite markers served to validate their utility for use on the *Chionoecetes* species group on the EBS shelf with at least four unique alleles identified for each species in the evaluation subset. All genotypes were double‐scored and a random selection of 8% of samples were re‐genotyped to screen for laboratory errors. Samples were also flagged for re‐genotyping due to perceived anomalies either in initial genotyping or in subsequent preliminary statistical analysis (*n* = 109). Genotypes with possible laboratory error were excluded, and genotypes of flagged samples were individually evaluated and either replaced with results from a second run or excluded from analysis.

The program GDA (Lewis and Zaykin 2001) was used to evaluate marker performance by estimating observed and expected heterozygosity and the inbreeding coefficient (*F*_IS_) for each marker and testing for deviation from Hardy–Weinberg equilibrium (HWE) and pairwise independence (linkage equilibrium). All five microsatellites revealed polymorphisms in snow crab (12–31 alleles per locus). However, there were many instances of alleles that differed by only one or two base pairs and, due to the high frequency of some alleles, the effective numbers of alleles were relatively low, especially for *Co36‐nfrdi* (Table 1). Of greater concern, loci *Co34‐nfrdi* and *EC0106* demonstrated significant deviation from HWE (*p* < 0.001 for each locus) and heterozygote deficiency (*F*_IS_ = 0.277 and *F*_IS_ = 0.161, respectively), consistent with the presence of null alleles. Additionally, the peaks on the electropherograms for *ECO106* were abnormal and difficult to score. Results from *Co34‐nfrdi* and *EC0106* were excluded, and genotypes from the remaining markers, *Co17‐nfrdi*, *Co36‐nfrdi*, and *Cop2*, were used in data analysis. Although these three markers followed HWE expectations, linkage disequilibrium was detected for the pair *Co36‐nfrdi*/*Cop2* (*p* = 0.004). The program GERUD 2.0 (Jones 2005) was used to calculate marker exclusion probabilities assuming one known parent genotype. The combined exclusion probability for these three markers was high (0.947).

**TABLE 1:** Five microsatellite markers were evaluated using genotypes of 33 snow crab females collected from the eastern Bering Sea in 2009 to 2016.

Locus	*N*	*N*_A_	*N*_eff_	Freq_max_	*H*_O_	*H*_E_	*F*_IS_	*p*_excl_
*Co17‐nfrdi* ^a^	33	12	5.7	0.273	0.818	0.824	0.023	0.660
*Co36‐nfrdi* ^a^	33	13	3	0.561	0.727	0.664	−0.080	0.490
*Cop2* ^a^	32	14	6.2	0.312	0.813	0.840	0.048	0.696
*Co34‐nfrdi*	33	15	7.3	0.273	0.636	0.863	0.277	0.735
*EC0106*	33	31	23.9	0.091	0.818	0.958	0.161	0.999

*Note:* Summary statistics include the number of crab successfully genotyped (*N*), the observed number of alleles (*N*_A_), the effective number of alleles, computed as (1−*H*_E_)^−1^, the maximum allele frequency (Freq_max_), the observed heterozygosity (*H*_O_), the expected heterozygosity (*H*_E_), the inbreeding coefficient (*F*_IS_), and the exclusion probability with one known parent genotype (*p*_excl_).

^a^ Loci used in data analysis.

### 2.3 Maternal Lineage of Hybrids

Hybrids result from interspecies mating between snow and Tanner crabs at an unknown prior generation in their lineage. Merus samples from putative hybrids were used to confirm species identity and identify maternal lineage, i.e., the species of female that mated with a male congener. Spatial patterns in maternal lineage of hybrids were assessed among six regions defined by the intersection of three areas developed from dominant ontogenetic migration patterns of snow crab (northwest, central, and southeast areas; Parada et al. 2010) and two oceanographic domains of the continental shelf based on frontal zones that follow bathymetric contours (middle: 50–100 m and outer: 100–180 m; Stabeno et al. 2001; Figure 2). The proportions of hybrids with a snow crab maternal lineage were estimated for each sampled station (*n* = 67), with one to 35 hybrids contributing to each estimate. Sampled stations were distributed across years and regions (Table 2a). Estimates were averaged across stations in a region to provide annual regional proportions of hybrids originating from a snow crab maternal lineage. Mature female snow crab and Tanner crab abundances in the EBS were estimated using area‐swept methods (Zacher et al. 2023) and data from the NOAA EBS shelf bottom trawl survey (accessed via Alaska Fisheries Information Network, https://akfin.psmfc.org). The proportions of snow crab abundance relative to the combined abundance of mature female snow and Tanner crabs were calculated by region and year, allowing for comparison to the proportions of hybrids with a snow crab maternal lineage.

**TABLE 2:** (a) The numbers of stations where hybrids were sampled to assess maternal lineage (numbers of hybrids are provided in parentheses), (b) average proportions of hybrids with a snow crab maternal lineage, and (c) proportions of snow crab abundance to the combined abundance of mature female snow and Tanner crabs.

(a)									
Region	2009	2010	2011	2012	2013	2014	2015	2016	Totals
NW outer	1 (1)	2 (10)	2 (19)	3 (27)	1 (15)	0	0	1 (12)	10 (84)
NW middle	0	0	1 (2)	0	1 (7)	0	0	0	2 (9)
CEN outer	3 (8)	3 (13)	0	0	0	0	1 (3)	0	7 (24)
CEN middle	8 (23)	1 (2)	1 (3)	4 (14)	3 (9)	1 (2)	1 (1)	0	19 (54)
SE outer	0	0	1 (4)	1 (14)	0	0	2 (7)	1 (1)	5 (26)
SE middle	8 (47)	1 (1)	6 (10)	1 (4)	6 (11)	0	2 (21)	0	24 (94)
Totals	20 (79)	7 (26)	11 (38)	9 (59)	11 (42)	1 (2)	6 (32)	2 (13)	67 (291)

(b)									
Region	2009	2010	2011	2012	2013	2014	2015	2016	Averages
NW outer	1.00	1.00	0.97	1.00	0.87	na	na	0.92	0.97
NW middle	na	na	0.00	na	0.86	na	na	na	0.43
CEN outer	0.00	0.21	na	na	na	na	1.00	na	0.23
CEN middle	0.21	0.00	1.00	1.00	0.92	0.50	1.00	na	0.57
SE outer	na	na	0.00	0.14	na	na	0.70	0.00	0.31
SE middle	0.00	0.00	0.36	0.25	0.38	na	0.36	na	0.23

(c)									
Region	2009	2010	2011	2012	2013	2014	2015	2016	Averages
NW outer	0.98	1.00	1.00	1.00	1.00	0.98	0.94	0.86	0.99
NW middle	1.00	1.00	1.00	1.00	1.00	1.00	1.00	1.00	1.00
CEN outer	0.98	0.98	1.00	1.00	1.00	1.00	1.00	0.99	0.99
CEN middle	0.97	1.00	0.99	0.94	0.87	0.94	0.98	0.93	0.97
SE outer	0.24	0.58	0.06	0.26	0.57	0.89	0.89	0.84	0.76
SE middle	0.71	0.32	0.56	0.55	0.60	0.64	0.54	0.71	0.60

*Note:* Data were summarized by year and region, defined by the intersection of three areas (NW: Northwest, CEN: Central, SE: Southeast) and two oceanographic domains (middle: 50–100 m, outer: 100–180 m; Figure 2).

### 2.4 Mates Detected in Female Sperm Reserves

Samples of seminal receptacle contents were used to evaluate the mating history of primiparous and multiparous putative snow crab collected across multiple years (2007–2016) and areas (northwest, central, and southeast) (Table A3 in Appendix A). Crab had been processed fresh (*n* = 778), within a few days of death (*n* = 151), or after being frozen (*n* = 62). Because female merus tissue was unavailable from the archive collection, a sample of epithelial tissue from the seminal receptacle was used to determine the female genotype. Seminal receptacles were separated into their constituent components of female epithelial tissue and the contents containing male ejaculate material. Contents were bisected longitudinally, with one half returned to the ADF&G sample archive. The other half was either left whole or sectioned into multiple subsamples to reflect individual ejaculate layers based on visual evaluation of the contents and reference to the number of ejaculate layers estimated for the complementary right seminal receptacle. When data were unavailable from the right side of a pair, contents from the left side were divided into half if sufficient material was present or else collected as a single subsample. This procedure partitioned seminal receptacle contents into one (*n* = 566), two (*n* = 413), or three (*n* = 12) subsamples for each crab (Table A4 in Appendix A).

**FIGURE 3:**
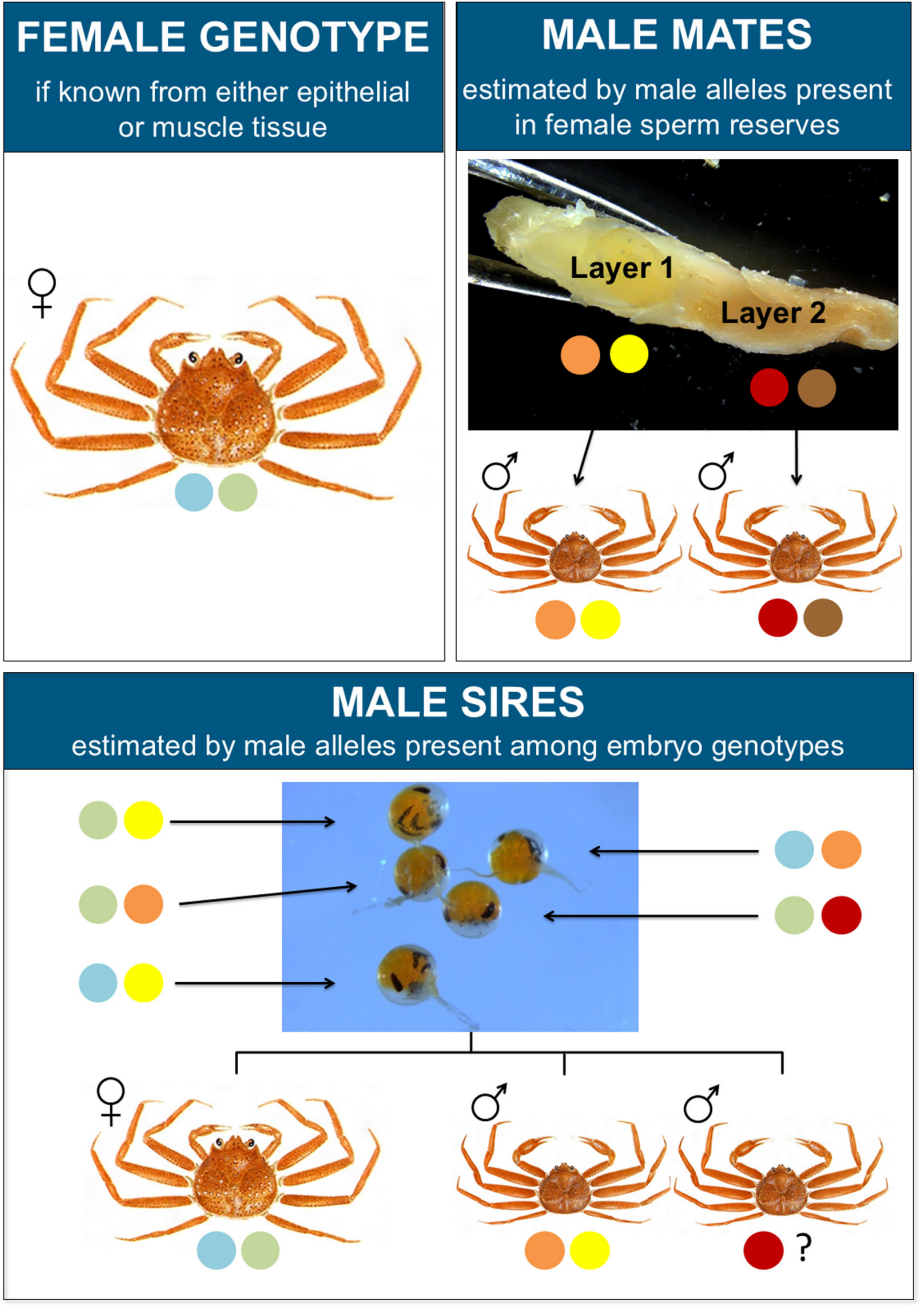
Conceptual framework for the use of molecular markers to estimate male mates present in female sperm reserves and male sires present among embryo genotypes, after accounting for the female genotype. An individual inherits two alleles, one from each parent, at each chromosomal location. The colored dots indicate the hypothetical alleles for each individual at a single molecular marker. A combination of markers was used to accomplish study objectives.

The mating histories of female snow crab were characterized by the minimum number of mates detected in their sperm reserves, using microsatellite data from both female tissue and seminal receptacle contents (Figure 3). After verification of female putative species identifications using SNP genotypes, microsatellite data were screened to include only instances for which all available samples from the seminal receptacle contents had been successfully scored (i.e., were non‐null) at a minimum of two markers. Alleles that matched the female genotype were identified, and an average of 4.9 female alleles were present in seminal receptacle contents (out of six alleles possible across the three markers, with homozygote genotypes counted as two alleles; *n* = 923 crab). To account for female contamination, female alleles were removed from the pooled genotypes at each marker when known, and otherwise the number of alleles was reduced by two.

After pooling alleles among seminal receptacle content samples from a female, the minimum number of mates was estimated as half the maximum number of non‐female alleles detected at two or more markers, rounded up to the nearest integer if the number of alleles was uneven. Results supported by more than one marker were used to account for uncertainty in allele identification resulting from pooled genotypes, given each seminal receptacle sample could include the female genotype along with one or more male genotypes. Because genotypes from each sample were determined independently, it is possible a single allele was scored as similar but different allele variants among multiple samples from a crab. This estimate may underestimate mates because it assumes males are heterozygotes and that no alleles are shared between the males or with the female. Additionally, the estimate reflects males that contributed sperm remaining in the seminal receptacles and does not include males that contributed sperm that was fully expended during fertilization of the current or prior clutches.

The minimum numbers of mates were evaluated across life history stages using a chi‐squared test. The species of mates in female sperm reserves was determined using SNP genotypes for each sample of seminal receptacle contents. The occurrence of interspecies mating was evaluated using chi‐squared tests across life history stages, collection areas, and the presence or absence of fresh ejaculate. For instances where the number of ejaculate layers was available from the right seminal receptacle, values were compared to the minimum number of mates from the complementary left side. These comparisons were used to evaluate whether the number of ejaculate layers provides an effective proxy for the number of mates.

### 2.5 Paternity and Presence of All Sires in Female Sperm Reserves

Ovigerous female snow crab were collected in 2017 and 2018 to evaluate the paternity of embryos in brooded clutches and to determine whether all sires remained present in female sperm reserves. Primiparous and multiparous crab were collected to meet sampling goals (i.e., 50 females per life history stage per area) during the NOAA EBS shelf bottom trawl survey in July of both years. Despite attempts over 2 years, no collections were obtained from the southeast area, so collections represent snow crab in the central and northwest areas. Crab were transported to the ADF&G seawater laboratory in Kodiak and held live for an extended duration (i.e., 4–8 months) until embryo development progressed to an eyed stage (i.e., stage 10 or greater, Moriyasu and Lanteigne 1998). Crab were processed fresh (*n* = 99) or within a day of death (*n* = 1) following methods developed for the ADF&G long‐term study. Reagent ethanol was used to preserve the left seminal receptacle and the abdominal flap removed with the brooded clutch intact. Females had clutch fullness scores of half full (*n* = 19), three‐quarters full (*n* = 47), or full (*n* = 34). The female genotype was determined using either a sample of merus muscle tissue or seminal receptacle epithelial tissue.

To determine how many embryos to sample from brooded clutches, the model PrDM (Neff and Pitcher 2002) was used to estimate the probability of detecting multiple paternity. The power to detect multiple paternity is a function of marker polymorphism, the number of embryos genotyped, the number of sires, and their relative contribution to clutch fertilization. Detection power increases with the number of embryos used and decreases with increasing skew in relative paternal contribution. Results suggested a sample size of 20 embryos per clutch would provide adequate power (0.82) to detect multiple paternity, assuming the most extreme paternal skew reported for snow crab (i.e., 92:8, Sainte‐Marie et al. 2008). To account for potential embryo stratification within the clutch (Sainte‐Marie and Carrière 1995), a minimum of 20 individual embryos were collected from throughout the clutch using a standardized three‐dimensional sampling pattern.

Paternity was characterized by the minimum number of sires contributing to the paternity of embryos sampled from brooded clutches (Figure 3). Maternal species identifications were confirmed using SNP genotypes obtained from female tissue. The *ITS* marker was used to determine embryo species, and thus infer sire species, since the maternal species was already known. Maternal and embryo microsatellite data were screened to exclude incomplete, inconsistent, or spurious results. All microsatellite marker genotypes were required to be successfully scored (i.e., were non‐null) at a minimum of two of the three markers used in data analysis. Individual embryo genotypes were further subject to two additional criteria: (1) they must exhibit at least one non‐maternal allele across the three markers, and (2) they must share at least one maternal allele at a minimum of two of the three markers. Any non‐null embryo markers that were incompatible with the maternal genotype (i.e., lacking maternal alleles) were set to null and treated as scoring failures in data analysis. These data screening measures resulted in reduced embryo samples sizes for some females.

After pooling alleles among embryos sampled from a clutch, the minimum number of sires was estimated as half the maximum number of paternal alleles detected at any marker, rounded up to the nearest integer if the number of alleles was uneven. For each embryo, one allele was assigned as a maternal allele and one as a paternal allele. If an embryo genotype matched the female genotype or was homozygous for one of the female alleles, an allele was attributed to the sire even if it matched the female genotype. This estimate may underestimate sires because it assumes males are heterozygotes. The minimum numbers of sires were confirmed using the program GERUD 2.0, which also provided putative sire genotypes. In instances of multiple paternity, the program additionally provided a list of possible sire genotypes together with the number of embryos compatible with each sire ordered by likelihood. The scenario with the highest likelihood was reported.

The minimum number and species of mates detected in female sperm reserves were estimated using the same methods described for other study objectives. Seminal receptacle contents were unavailable for females with empty seminal receptacles (*n* = 3) and otherwise were represented in one (*n* = 78) or two (*n* = 19) samples from each crab. The minimum numbers of sires and minimum numbers of mates were evaluated between life history stages using chi‐squared tests. The presence of sires in female sperm reserves was assessed by examining whether putative sire alleles were present in seminal receptacle contents. Specifically, a sire genotype was recorded as present if its alleles were among the pooled non‐female alleles in seminal receptacle contents for at least two markers. Results were summarized as the presence or absence of all sires in female sperm reserves, with absence indicating sperm from a male was fully expended during fertilization of the brooded clutch. The proportions of instances where all sires were present in female sperm reserves were compared between paternity groups (i.e., single or multiple) and life history stages using two‐sample tests of proportions. Average seminal receptacle load was compared between life history stages using a two‐sample t‐test. Average clutch fullness score was compared between females with or without the presence of all sires in their sperm reserves within each life history stage using a Wilcoxon rank‐sum test.

## 3 Results

### 3.1 Maternal Lineage of Hybrids

Putative species identifications were confirmed for most hybrids (*n* = 296), except a few identified as snow crab (*n* = 12) or Tanner crab (*n* = 2) and those excluded due to genotyping error (*n* = 2). Two hybrids identified in ancillary genotyping of putative snow crab and Tanner crab were added to the dataset. *16S* SNP genotypes were determined for most of the confirmed hybrids (*n* = 291). The average proportion of hybrids with a snow crab maternal lineage varied across regions, with predominantly snow crab maternal lineage in the northwest outer region, predominantly Tanner crab maternal lineage in both southeast regions as well as the central outer region, and a mixture of maternal lineages in the northwest middle and central middle regions (Table 2b). Snow crab comprised the majority of the surveyed population of mature snow and Tanner crab females during the years examined, especially in the northwest and central areas (Table 2c).

### 3.2 Mates Detected in Female Sperm Reserves

**FIGURE 4:**
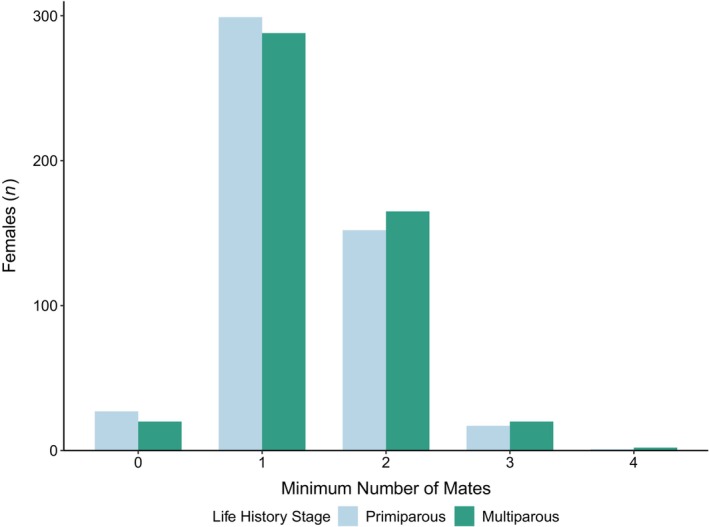
The numbers of female snow crab by the minimum number of mates represented in their sperm reserves and by life history stage.

Putative species identifications were confirmed for most snow crab, except a few identified as hybrids (*n* = 3). Most females showed evidence of a single mate in their sperm reserves (59%; Figure 4). Polyandry was detected less frequently (36%) and was typically associated with the presence of sperm from two mates (32%), though some females had sperm from up to four mates. There were some females (5%) with no mates detected (i.e., no residual sperm present). The proportions of females corresponding to each estimate of minimum number of mates were similar across life history stages (*p* = 0.54, with counts combined for three and four mates).

SNP genotypes were evaluated for most females, except those associated with genotyping errors across all seminal receptacle samples (*n* = 20). Most snow crab females mated only with snow crab males (*n* = 912 crab with *n* = 1320 seminal receptacle samples), based on *ITS* SNP genotypes present in female sperm reserves. Interspecies mating was detected in 2% of snow crab females (*n* = 17 crab with *n* = 26 seminal receptacle samples). Among these females, those with results available for multiple seminal receptacle samples (*n* = 7) had multiple *ITS* genotypes detected, including the presence of both interspecific and conspecific mates (Table 3). Detection of the *ITS* genotype C/G could either indicate a hybrid mate or a sample containing multiple individuals including a Tanner crab mate along with either a snow crab mate or contamination from the female snow crab's seminal receptacle. Interspecies mating was detected more frequently for multiparous females (*n* = 14, *p* = 0.007) and females from the southeast area (*n* = 14, *p* < 0.001; Table 3). The occurrence of interspecies mating was similar between females with or without the presence of fresh ejaculate (*p* = 0.63).

**TABLE 3:** Mating history for 17 female snow crab with genetic evidence of interspecies mating.

Life history stage	Area	Ejaculate layer age	Seminal receptacle sample location			
			Single	Proximal	Intermediate	Distal
Primiparous	CEN	Fresh	C/G			
Primiparous	SE	Fresh	C/G			
Primiparous	SE	Fresh	C/G			
Multiparous	NW	Fresh	C/G			
Multiparous	CEN	Fresh + old		C/G		C/C
Multiparous	SE	Fresh + old		G/G		C/G
Multiparous	SE	Fresh	C/C			
Multiparous	SE	Fresh	C/G			
Multiparous	SE	Fresh	C/G			
Multiparous	SE	Fresh	C/G			
Multiparous	SE	Fresh	C/G			
Multiparous	SE	Fresh		G/G		C/G
Multiparous	SE	Fresh		NA		C/C
Multiparous	SE	Fresh		C/G		G/G
Multiparous	SE	NA		G/G		C/G
Multiparous	SE	NA		C/G		C/C
Multiparous	SE	Fresh		G/G	C/G	C/G

*Note:* Each row represents an individual female. Details include life history stage, area of collection (NW: Northwest, CEN: Central, SE: Southeast), and ejaculate layer age, indicating contributions gained in the most recent mating season (fresh), a prior mating season (old), or unknown (NA). Genotypes are provided for each seminal receptacle sample, with sample location noted as single or as the proximal, intermediate, and distal locations relative to the seminal receptacle opening when multiple samples were collected. Mate species is based on the nucleotides present at the *ITS* marker and indicate snow crab (both guanine: G/G) or Tanner crab (both cytosine: C/C). A combination of guanine and cytosine nucleotides (C/G) could indicate either a hybrid mate or a sample containing multiple individuals including a Tanner crab mate and either the snow crab female or a snow crab mate. Seminal receptacle samples associated with genotyping error are noted as NA.

Comparison of results from both sides of paired seminal receptacles showed that the number of ejaculate layers corresponded to the minimum number of mates for 58% of females (Table 4). The majority of females (98%) had one or two estimated ejaculate layers. For females with a single ejaculate layer, the minimum number of mates was higher in 26% of cases. In contrast, for females with two ejaculate layers, the minimum number of mates was lower in 47% of the cases.

**TABLE 4:** The numbers of snow crab females by minimum number of mates and number of ejaculate layers.

Minimum number of mates	Number of ejaculate layers					Totals
	0	1	2	3	NA	
0	0	35	7	0	5	42
1	4	387	151	3	42	545
2	1	142	150	9	15	302
3	0	5	26	4	2	35
4	0	0	1	1	1	2
Totals	5	569	335	17	65	991

### 3.3 Paternity and Presence of All Sires in Female Sperm Reserves

Putative species identifications were confirmed for all snow crab females. SNP genotypes were determined for most embryos, except those associated with genotyping error (*n* = 9) or missing sample (*n* = 1). Almost all embryos were identified as snow crab (*n* = 2045), except one identified as a hybrid. After screening microsatellite genotypes (Table A5 in Appendix A), data from 1644 embryos were used to estimate the minimum number of sires contributing to embryos in brooded clutches of 43 primiparous and 46 multiparous females, with a minimum of seven embryos per clutch. All embryos had snow crab sires and most clutches had single paternity (82%). Multiple paternity, with dual paternity (at minimum), was detected in 16 of 89 brooded clutches (Table 5). In instances of dual paternity, contributions were always skewed toward one of the two sires, with an average of 78% of embryos from a clutch resulting from the dominant sire (Table 6). Although not statistically significant (*p* = 0.22), the frequency of multiple paternity for multiparous females (0.24) was nominally twice that for primiparous females (0.12; Table 5).

**TABLE 5:** The numbers of snow crab females by life history stage, displayed by minimum number of sires, minimum number of mates, and whether all sires were present in female sperm reserves.

Life history stage	Minimum number of sires	*n*	Minimum number of mates					All sires present in sperm reserves	
			0	1	2	3	Totals	True	False
Primiparous	1	38	4^a^	24	8	1	37	23	14^a^
Primiparous	2	5	1^a^	4	0	0	5	1	4^a^
Multiparous	1	35	4	20	11	0	35	27	8
Multiparous	2	11	1	6	4	0	11	6	5
Totals		89	10^a^	54	23	1	88	57	31^a^

^a^ Three females with empty seminal receptacles included.

**TABLE 6:** The numbers of embryos genetically compatible with and likely attributable to each of two sires for 16 broods with dual paternity and the proportions of embryos contributed by the dominant sire.

Embryos (*n*)	Sire 1	Sire 2	Sire 1 contribution
20	12	8	0.60
20	18	2	0.90
20	19	3	0.86
20	19	3	0.86
19	13	6	0.68
19	17	2	0.89
19	15	4	0.79
19	17	5	0.77
19	16	3	0.84
19	18	2	0.90
18	16	2	0.89
18	17	5	0.77
18	13	5	0.72
14	8	6	0.57
8	7	3	0.70
7	5	2	0.71
Averages	14	4	0.78

*Note:* Instances where the sum of embryos attributed to sires is greater than the embryo sample size indicates the presence of embryos that were genetically compatible with both sires. Results reflect scenarios with the highest likelihood obtained from GERUD 2.0 (Jones 2005).

The power to detect multiple paternity was generally high for the three microsatellite makers used in this study across the range of embryo sample sizes available for analysis (range: 7–29), assuming relative paternal contributions of two sires as 50:50, 75:25, and 90:10 (Table 7). Probabilities of detection ranged from 0.33 for highly skewed paternal contributions and a sample size of five embryos to 0.99 for equal paternal contributions and a sample size of 30 embryos (Neff and Pitcher 2002). While the risk of failing to detect multiple paternity was highest with smaller embryo sample sizes, multiple paternity was still detected in the smallest sample size in this study (Table 6).

**TABLE 7:** The probability of detecting multiple paternity for the three microsatellite markers used in this study, encompassing the range of embryo sample sizes realized in this study (i.e., 7–29) and different relative paternal contributions of two sires (model PrDM, Neff and Pitcher 2002).

Relative paternal contribution	Embryos (*n*)			
	5	10	20	30
50:50	0.80	0.96	0.99	0.99
75:25	0.63	0.89	0.97	0.98
90:10	0.33	0.58	0.82	0.91

After screening microsatellite genotypes (Table A5 in Appendix A), data from 100 samples of seminal receptacle contents obtained from 85 females were used to determine whether all sires were present in female sperm reserves. All samples of seminal receptacle contents indicated snow crab mates based on SNP genotypes. The most common estimate of the minimum number of mates contributing to female sperm reserves was one (61%), followed by two (26%; Table 5). Females with no mates detected (11%) included females with empty seminal receptacles. The minimum numbers of mates were similar between life history stages (*p* = 0.50, with counts combined for two and three mates; Table 5).

**FIGURE 5:**
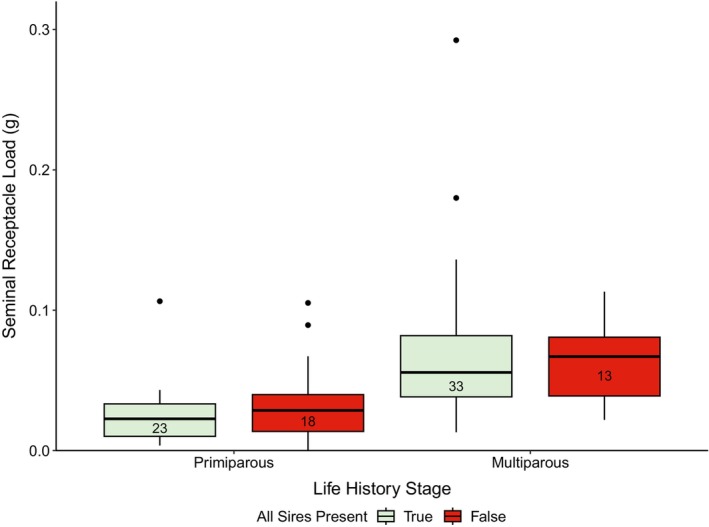
Box plots of seminal receptacle load (g) by life history stage and whether all sires were present in female sperm reserves. No differences were detected in seminal receptacle load between females with or without the presence of all sires in their sperm reserves within each life history stage. The saple size is provided inside each box.

The genotypes of all sires were detected among non‐maternal alleles in the seminal receptacle contents for the majority of females (57 of 88 females; Table 5). The presence of all sires in female sperm reserves was more common in cases of single paternity (69%) than multiple paternity (44%), and more frequent for multiparous females (72%) than primiparous females (57%), though neither paired difference was statistically significant (*p* = 0.098 and *p* = 0.23, respectively). While average seminal receptacle load was greater in multiparous females than in primiparous females, no significant differences were detected within each life history stage between females with or without the presence of all sires in their sperm reserves (*p* = 0.61 for primiparous females and *p* = 0.99 for multiparous females; Figure 5). Similarly, although average clutch fullness was higher in multiparous females (5.7) than in primiparous females (4.7), no significant differences were observed within each life history stage between females with or without the presence of all sires in their sperm reserves (*p* = 0.23 for primiparous females and *p* = 0.174 for multiparous females).

## 4 Discussion

In summary, results from this study suggest that interspecies mating has minimal impact on snow crab female reproductive potential in the EBS, given the low incidence of snow crab females mating with congeners and the observation that hybrids can result from either snow crab or Tanner crab females. In addition, snow crab mating opportunities in the EBS may be limited, as indicated by the high proportion of females with sperm from only one male. While most females brooded clutches sired by a single male, dual paternity was detected in about one in five females, and more than one‐third of females lacked all sires' genotypes in their sperm reserves. These findings suggest that some females fully utilize sperm from some males during fertilization of the brooded clutch. As stored sperm is utilized, females may need to remate to avoid sperm depletion and ensure the fertilization of future clutches.

### 4.1 Genetic Markers

Although the microsatellite loci used in this study were developed for snow crab, they were effective for their congeners. Similarly, Urbani, Sévigny, et al. (1998) amplified loci developed for snow crab (note, those loci were different than the ones used in this study), used them for multiple crab species, and observed polymorphism in Tanner crab for five out of six loci. Additionally, Kang, Park, Kim, Choi, et al. (2013) evaluated transferability of several microsatellite markers developed for snow crab, including *Co17‐nfrdi*, *Co36‐nfrdi*, and *Co34‐nfrdi*, across multiple crab species, and most loci (though notably not *Co34‐nfrdi*) amplified for their examined congener species (i.e., red snow crab, *C. japonicus*).

### 4.2 Maternal Lineage of Hybrids

The results of this study on the maternal lineage of hybrids provide a broader geographic scope compared to an earlier study, which suggested that hybrids primarily result from mating between snow crab females and Tanner crab males (Smith, Grant, and Seeb 2005). That finding was based on male hybrids caught during the commercial snow crab fishery in the central outer region (Christian Smith, personal communication). In contrast, our results from the same region show mostly Tanner crab maternal lineage, although there was high variability in maternal lineage across years in the central area (Table 2b). Smith, Grant, and Seeb's (2005) finding of hybrids originating from mating between female snow crab and male Tanner crab had appeal based on: (1) the occurrence of sexual size dimorphism in *Chionoecetes* spp., with males achieving larger sizes than females; (2) observations of other crustaceans displaying sexual size dimorphism indicate that males generally need to be larger than females to mate successfully; and (3) the fact that Tanner crab achieve larger sizes than snow crab. In another *Chionoecetes* hybrid in the East Sea (Sea of Japan), hybrids resulted only from unidirectional hybridization between females of the smaller species (red snow crab) and males of the larger species (snow crab) (Kim et al. 2012). While snow crab pairing does not appear to be physically limited for different‐sized pairs, there may be behavioral factors that favor larger males for successful pairing, either through male competition for access to females or female choice assertions (Sainte‐Marie et al. 2008). For instance, in Tanner crab, multiparous females of size 80–110 mm carapace width evaded or resisted mating with males of equal or smaller size (Adams 1985). Additionally, reproductive barriers may limit the production of offspring resulting from one direction of an interspecies cross. For example, out of 80 published cases of hybridization summarized by Wirtz (1999), 50 resulted only from interspecies mating in only one direction (male X and female Y but not male Y and female X), and multiple prezygotic and postzygotic reproductive barriers were discussed. The finding of bidirectional hybridization in this study indicates reproductive barriers are lacking between these species and that factors in addition to size dimorphism likely contribute to interspecies mating. These factors may include the relative abundance of each species by sex and size, which can vary greatly over time and space in the EBS (Zacher et al. 2023).

Further work is needed to determine whether hybrids are reproductively viable. Examination of alleles at multiple allozymes for putative snow and Tanner crabs suggested the introgression of genetic material between the species based on the presence of alleles associated with snow crab in Tanner crab collected from the EBS, but not in Tanner crab collected from multiple other locations (Merkouris, Seeb, and Murphy 1998). However, those alleles could have resulted from mutations within the EBS Tanner crab population, rather than as introgression from snow crab.

Although the location of sampled hybrids in the EBS and their maternal lineage were known, inferring where interspecies mating occurred is challenging. Larval advection and movement of hybrids between settlement and collection limit our ability to examine spatial processes associated with hybridization in the EBS. Spatial dynamics of snow and Tanner crabs in the EBS are complex; larval advection can transport newly hatched crabs to distant locations, typically northward (Parada et al. 2010; Richar et al. 2015), and snow crab undergo ontogenetic migration, often moving southwest across the continental shelf (Ernst, Orensanz, and Armstrong 2005). If subsequent generations of offspring from interspecies mating exist in the EBS, the time between the collection of an adult hybrid and its ancestral interspecific mating could vary greatly, from the development time of a single generation (i.e., 7–8 years, Ernst et al. 2012) to multiple generations.

Despite these limitations, we observed some spatial associations between hybrid maternal lineage and the relative abundance of mature female snow crab (Table 2). It is important to note that our reported proportions of maternal lineage may be biased, as we only sampled mature female hybrids and did not account for differences in hybrid abundance among stations within a region. The prevalence of a snow crab maternal lineage was highest in the northwest area, where the mature female population was almost entirely snow crab, and lowest in the southeast area, where a more mixed assemblage of snow and Tanner crabs were found (Table 2c). The central area may represent an intermediate mixing zone between the northwest and southeast areas, where maternal lineage was variable despite the low relative abundance of mature female Tanner crab during the study period.

Across the study objectives, misidentification of crabs based on morphological features occurred rarely, only 1% of the time (*n* = 19 crabs out of *n* = 1479 crabs), and involved hybrid identifications. Given the subjective nature of identifying hybrids by visual evaluation of morphological characters and the possible presence of F2 or later generation hybrids in the population (resulting from hybrids reproducing with other hybrids or backcrossing with snow crab or Tanner crab), the low error rate is noteworthy.

### 4.3 Mates Detected in Female Sperm Reserves

Results from this study indicate most primiparous snow crab in the EBS mated with one male, though they can mate with up to three males in 1 year. While a few females mated with up to four males, those observations likely reflect a combined mating history over multiple mating seasons. The observation of similar minimum numbers of mates between life history stages is noteworthy. For multiparous females, this could indicate either the full utilization of a mate's sperm during clutch fertilization combined with replacement during subsequent mating events or the detection of remaining sperm from a female's first mating season. The increased seminal receptacle load observed for multiparous females (Figure 5) supports the first explanation, because a decrease in seminal receptacle load would be expected if sperm were utilized for clutch fertilization without replenishment, but further work on this topic is warranted.

The minimum number of mates in this study were generally lower than results reported for snow crab in the Gulf of St. Lawrence, where estimates were based on the maximum allele count at a single marker and ranged from three to five mates (*n* = 7) (Urbani, Sainte‐Marie, et al. 1998) or one to five mates for primiparous females (*n* = 20) (Sainte‐Marie et al. 1999) and two to six mates for multiparous females (Sainte‐Marie et al. 2008). Studies on snow crab in the Gulf of St. Lawrence have shown the relative quantity of material stored in female sperm reserves and the intensity of polyandry during a female's first mating season are positively related to the relative abundance of large, hard‐shell adult males (Rondeau and Sainte‐Marie 2001; Sainte‐Marie, Sévigny, and Carpentier 2002).

Although observations of interspecies mating were limited, occurrences may indicate a lack of conspecific males at a local level during the mating season. Azuma et al. (2011) evaluated phylogenetic relationships among crabs in the *Chionoecetes* genus using mitochondrial DNA sequences and found that, while Tanner crab and snow crab were closely related, there was substantial divergence indicating well‐differentiated species. Females, which have a greater parental investment (e.g., caloric expenditure and invested time) than males, typically reject interspecific mates, except when conspecific males are rare (Wirtz 1999). Given our understanding of snow crab mating dynamics, the occurrence of interspecies mating among multiparous females was unexpected. We anticipated interspecies mating to be more common in primiparous females due to the lack of mate choice during a female's first mating season. The finding that interspecies mating was more frequently detected in multiparous females, most of whom had only fresh ejaculate (Table 3), suggests that these females may have actively chosen to mate outside their species. This could be due to a scarcity of conspecific males or metrics related to the relative fitness of available males, such as size. The occurrence of snow crab females with evidence of interspecies mating in the southeast area likely reflects the geographic distribution of Tanner crab, which is high in the southeast area and would result in an increased frequency of contact with male Tanner crab.

The number of ejaculate layers did not provide an effective proxy for the number of mates (Table 4). There are multiple reasons estimated ejaculate layers may overestimate mates, including the full utilization of sperm from a mate during clutch fertilization, as observed in 35% of females in this study. In these instances, seminal fluid from the contributing male may remain in the seminal receptacle, especially as DNA‐rich sperm tends to settle to the bottom of each ejaculate layer (Bernard Sainte‐Marie, DFO, personal communication). Additionally, one male can contribute multiple ejaculate layers, with repeated mating observed most frequently among smaller males (Sainte‐Marie, Sévigny, and Gauthier 1997). Most female snow crab had no more than two estimated mates and two estimated ejaculate layers in their sperm reserves. We advise the use of other indicators of recent mating success and presence of sperm reserves, including the presence of fresh ejaculate and relative spermathecal fullness (Webb et al. 2016; Webb and Bednarski 2010; Duluc, Sainte‐Marie, and Brêthes 2005).

### 4.4 Paternity and Presence of All Sires in Female Sperm Reserves

The finding of dual paternity in 18% of wild‐caught primiparous and multiparous females was higher than reported in previous studies of snow crab. Single paternity was detected for all primiparous snow crab in the Gulf of St. Lawrence, including both wild‐caught (*n* = 7; *n* = 5) and laboratory‐mated (*n* = 11) females (Urbani, Sainte‐Marie, et al. 1998; Sainte‐Marie et al. 1999). Dual paternity was detected in 10%–15% of wild‐caught multiparous females, with contributions primarily from one of the two sires (*n* = 20, examined for penultimate and last clutch) (Sainte‐Marie et al. 2008). However, our results may not be directly comparable to those studies, as we examined the genotypes of individual embryos rather than pooled embryos, allowing for greater detection of multiple paternity, especially in cases where alleles were shared between the mother and an unknown sire. For other crab species, variable paternity findings have been reported, including only single paternity for red king crab (*Paralithodes camtschaticus*, *n* = 24) (Vulstek et al. 2013) and brown crab (*Metacarcinus edwardsii*, *n* = 31) (Pardo et al. 2016) and multiple paternity detected in 34% of Dungeness crab (*M. magister*, *n* = 29) (Worton et al. 2010), 40% of mangrove land crab (*Ucides cordatus*, *n* = 10) (Baggio et al. 2011), and 80% of an intertidal porcelain crab (*Petrolisthes cinctipes*, *n* = 10) (Toonen 2004).

While all sire genotypes were typically observed in female sperm reserves, for 35% of females at least one sire was not present, indicating full utilization of ejaculate contributed from some males during fertilization of the clutch. For comparison, sire genotypes from clutches with single paternity were present in female sperm reserves of all wild‐caught primiparous snow crab in the Gulf of St. Lawrence (*n* = 7) (Urbani, Sainte‐Marie, et al. 1998). In this study, the presence of all sires in the sperm reserves was more common with single paternity than with multiple paternity, suggesting ejaculate from a male was likely fully expended during fertilization of clutches sired by multiple males. This aligns with the stratified nature of ejaculate layers (Urbani et al. 1998). Indicators of low sperm reserves have been associated with reduced fecundity for multiparous snow crab females (Webb et al. 2016). If full utilization of sperm from a male indicated low remaining sperm reserves, we would expect to see reduced seminal receptacle load and potentially reduced fecundity. However, no relationship was found between the presence of all sires in the sperm reserves and either seminal receptacle load (Figure 5) or clutch fullness score.

### 4.5 Future Research

Future research may focus on other brachyuran crabs, such as Tanner crab, to provide important insights into mating dynamics and paternity of embryos. Although snow crab appears to constitute one large, panmictic population throughout the EBS, Chukchi, and Beaufort Seas (Albrecht et al. 2014), allozymes indicate that genetic differences exist among Tanner crab populations in Southeast Alaska, Gulf of Alaska, and the EBS, including within the EBS (Merkouris, Seeb, and Murphy 1998). Moreover, separate Tanner crab stocks reside in geographically widely separated embayments throughout the Gulf of Alaska and Southeast Alaska. This multi‐stock structure provides an opportunity to examine factors affecting reproductive dynamics in Tanner crab in ways not available to the single snow crab stock. For instance, future studies could contrast implications of geography (e.g., broad continental shelf of the EBS versus isolated embayments), sex ratio, and fishing pressure on ability of reproductively active male and female Tanner crabs to associate with one another annually and its effects on features such as clutch size, presence of fresh ejaculate, number of males contributing to female sperm reserves, and multiple paternity (e.g., Webb and Bednarski 2010).

### 4.6 Fishery Management Implications

Findings from this study demonstrate most snow crab females in the EBS mate with snow crab males, and the incidence of hybrids resulting from interspecies mating by snow crab females is low. These results suggest that impacts on snow crab stock productivity due to interspecies mating are not sufficient to be a pressing fishery management concern. The production of hybrids may become less of a fishery management issue in the future with climate change, as snow crab habitat and distribution in the EBS has been shrinking and shifting northward with increased temperatures and the reduced extent of the cold pool (Fedewa et al. 2020; Mueter and Litzow 2008). Recruitment of Tanner crab is reliant on local retention (Richar et al. 2015). The latitude of Tanner crab distribution was not correlated with environmental variables, and projections of productivity under forecasted climate change suggest their distribution may shift offshore, not northward (Szuwalski et al. 2021). As a result of the different responses between these species to changing environmental conditions, we anticipate that the degree of overlap and the extent of hybridization between the two species will decline in the future.

Our finding of few mates detected in female sperm reserves of EBS snow crab does raise some fishery management concerns, as it may indicate limited mating opportunities, which may result in limited stored sperm available to fertilize subsequent clutches without remating. Previous research showed female sperm reserves observed in the EBS were low relative to those observed in the Gulf of St Lawrence (Slater, MacTavish, and Pengilly 2010). Our results showed the extent of polyandry for EBS snow crab was also low relative to Gulf of St Lawrence snow crab. After a 3‐year period of heavy exploitation, seminal receptacle loads of primiparous females off Newfoundland, Canada, were low relative to long‐term monitoring sites in the northern Gulf of St. Lawrence but were similar to those observed in the EBS (Baker, Mullowney, and Sainte‐Marie 2022). Based on their findings, Baker, Mullowney, and Sainte‐Marie (2022) expressed biological concerns about the potential for sperm limitation and emphasized the importance of maintaining conservative exploitation rates that allow sufficient numbers of large adult male snow crab to preserve stock reproductive capacity. Likewise, the low extent of polyandry for EBS snow crab accentuates the importance of maintaining sufficient large mature males to ensure population renewal despite the large male‐only fishery that reduces the abundance of large, hard‐shell adult males available for mating. In association with an apparent mass mortality event leading to the loss of ~10 billion snow crab during 2018–2021 (Szuwalski et al. 2023), the percentage of mature females with clutches that were three‐quarters full or full declined from 87% in 2019 to 60% in 2021 to 40% in 2022 (Zacher et al. 2023). This reduction in an index of reproductive success, coupled with the lowest observed biomasses of both mature male and mature female snow crab (Zacher et al. 2023), heighten concerns for conservation to avoid further depleting reproductive potential.

Moreover, we feel that there is a compelling case for spatially explicit fishery management. The increasingly patchy, shrinking, and disparate distributions of mature males and mature females in the EBS observed during recent trawl surveys (Zacher et al. 2023) likely pose challenges to successful reproduction. A spatially explicit fishery management plan should be considered to help safeguard that the fishery does not depress male–female sex ratios to an extent that they trigger reproductive failures. Tagging studies and analysis of fishery and onboard observer data are needed to provide crucial information on crab movements to inform the development of such a spatial plan. Currently, the snow crab catch limit is set annually EBS‐wide, however the fishery concentrates in areas of greatest abundance of legal males. As climate change continues to affect snow crab distributions, it will be increasingly important to monitor area‐specific measures of reproductive success; spatiotemporal variation in the degree of polyandry, multiple paternity, and sex ratio indices could be associated with fishing intensity (e.g., Gosselin, Sainte‐Marie, and Bernatchez 2005). Finally, a spatially explicit fishery management plan should be informed by a spatially explicit length‐based stock assessment. Such an assessment would also help form the basis of a management strategy evaluation of harvest policy (e.g., Heller‐Shipley et al. 2021).

## Author Contributions

**Laura M. Slater:** conceptualization (equal), data curation (equal), formal analysis (equal), funding acquisition (equal), investigation (lead), methodology (lead), project administration (equal), resources (equal), supervision (equal), visualization (equal), writing – original draft (lead), writing – review and editing (lead). **William Gaeuman:** data curation (equal), formal analysis (equal), investigation (supporting), methodology (supporting), software (lead), visualization (equal), writing – original draft (supporting), writing – review and editing (supporting). **Wei Cheng:** methodology (supporting), project administration (equal), supervision (equal), writing – original draft (supporting), writing – review and editing (supporting). **Gordon H. Kruse:** conceptualization (equal), funding acquisition (equal), supervision (equal), writing – review and editing (supporting). **Christopher Habicht:** project administration (equal), resources (equal), writing – review and editing (supporting). **Douglas Pengilly:** conceptualization (equal), funding acquisition (equal), methodology (supporting), writing – review and editing (supporting).

## Conflicts of Interest

The authors declare no conflicts of interest.

### Open Research Badges

This article has earned Open Data, Open Materials and Preregistered Research Design badges. Data, materials and the preregistered design and analysis plan are available at https://doi.org/10.24431/rw1k6cq.

## Data Availability

Data generated and used for this study are available in the DataONE Research Workspace repository (https://doi.org/10.24431/rw1k6cq).

## Appendix A

**TABLE A1:** Details on the eight candidate microsatellite markers evaluated for this study, including locus, repeat motifs, numbers of alleles, and references of marker development.

Locus	Repeat motifs	Alleles (*n*)	References
Used in data analysis			
*Co17‐nfrdi*	CA(12)	22	Kang, Park, Kim, Ko (2013), Kang, Park, Kim, Choi, et al. (2013)
*Co36‐nfrdi*	CA(8)	24	Kang, Park, Kim, Ko (2013), Kang, Park, Kim, Choi, et al. (2013)
*Cop2*	CA(6), CA(8), CA(16)	24	Puebla, Parent, and Sévigny (2003)
Amplified but not used in data analysis			
*Co34‐nfrdi*	CA(13)	23	Kang, Park, Kim, Ko (2013), Kang, Park, Kim, Choi, et al. (2013)
*EC0106*	AC(21)	25	An, Jeong, and Park (2007)
Failed to amplify			
*Cop4‐1*	GA(29)	20	Urbani, Sévigny, et al. (1998)
*Cop24‐3*	GACA(15)	13	Urbani, Sévigny, et al. (1998)
*Cop77*	AG(37), CAGA(7), AG(5)	46	Puebla, Parent, and Sévigny (2003)

*Note:* The first five markers were successfully amplified, and the first three markers were used in data analysis.

**TABLE A2:** Details on the five microsatellite loci that amplified in our study, including forward and reverse (5′–3′) primers, GenBank accession numbers, and PCR thermal profiles.

Locus	Forward and reverse primers (5′–3′)	GenBank	Thermal profile
*Co17‐nfrdi*	F: CCAGTTAAGTCACCCGAAATAA		95°C/5 min; 36 cycles of (94°C/30 s + 58°C/45 s + 72°C/1 min); 72°C 10 min
	R: GGTCTCGACGGAATCAATAATA		
*Co36‐nfrdi*	F: GTATTCCTCTTCGTAAACACGC		95°C/5 min; 36 cycles of (94°C/30 s + 58°C/45 s + 72°C/1 min); 72°C 10 min
	R: TGCGAATATTCTGTTGCATTAC		
*Cop2*	F: CAATATCTCTCTTTGTACAGTTAGA	AY332734	95°C/5 min; 39 cycles of (94°C/30 s + 54°C/45 s + 72°C/1 min); 72°C 10 min
	R: CGTCACTCGCCATCCACCAA		
*Co34‐nfrdi*	F: GTGTAGCTATGGAACGTGATGA		95°C/5 min; 36 cycles of (94°C/30 s + 58°C/45 s + 72°C/1 min); 72°C 10 min
	R: CAAAAATTTGCAAGAGGAAAAG		
*EC0106*	F: GCCAACACTAAGCACTAAGGA	DQ252369	95°C/5 min; 38 cycles of (94°C/30 s + 59°C/45 s + 72°C/1 min); 72°C 10 min
	R: CGGAATGATACCAAGGTCTAT		

**TABLE A3:** The numbers of snow crab used to assess mating history in female sperm reserves by year and by area (NW: Northwest, CEN: Central, SE: Southeast) for (a) primiparous and (b) multiparous females.

(a)											
Area	2007	2008	2009	2010	2011	2012	2013	2014	2015	2016	Totals
NW	12	14	6	8	24	11	9	19	18	22	143
CEN	12	40	22	13	12	16	6	24	33	25	203
SE	2	11	0	2	36	42	24	27	6	0	150
Totals	26	65	28	23	72	69	39	70	57	47	496

(b)											
Area	2007	2008	2009	2010	2011	2012	2013	2014	2015	2016	Totals
NW	4	1	0	4	24	49	20	46	10	7	165
CEN	3	7	12	50	24	23	9	6	16	15	165
SE	3	0	11	0	3	9	21	45	58	15	165
Totals	10	8	23	54	51	81	50	97	84	37	495

**TABLE A4:** The numbers of samples collected from left seminal receptacle contents relative to the number of ejaculate layers estimated in the complementary right seminal receptacle (NA indicates data were unavailable).

Samples of seminal receptacle contents from the left side (*n*)	Number of ejaculate layers in the right side					Totals
	0	1	2	3	NA	
1	3	545	11	0	7	566
2	2	24	324	5	58	413
3	0	0	0	12	0	12
Totals	5	569	335	17	65	991

**TABLE A5:** The numbers of samples excluded during the screening process of microsatellite genotypes for the determination of paternity and the presence of all sires in female sperm reserves, by reason for exclusion and sample type. The numbers of samples used in analysis are also provided.

Reason for exclusion	Females (*n*)	Embryos (*n*)	Seminal receptacle contents (*n*)	Totals
Sample missing	0	1	1	2
Laboratory error	0	190	0	190
Genotyping error at 2+ markers	2	104	0	106
Exclusion of associated samples	9	40	15	64
Assumed genotyping error (no maternal allele)	0	2	0	2
No non‐maternal alleles (possible unfertilized embryo or female tissue contamination)	0	75	0	75
Total samples excluded	11	412	16	439
Total samples used in analysis	89	1644	100	1833
